# Neoadjuvant Chemoradiotherapy for Locally Advanced Rectal Cancer Using Infusional Gemcitabine: Immune Cell Infiltration Analysis and Updated Survival

**DOI:** 10.3390/cancers17243963

**Published:** 2025-12-12

**Authors:** Shouki Bazarbashi, Hadeel AlManea, Ali Aljubran, Ahmed Alzahrani, Ali Alqahtani, Fahad Almugbel, Muhammad Shahzad Rauf, Hazem Ghebeh

**Affiliations:** 1 Cancer Center of Excellence, King Faisal Specialist Hospital & Research Centre, Riyadh 11211, Saudi Arabia; bazarbashi@kfshrc.edu.sa (S.B.); ajubran@kfshrc.edu.sa (A.A.); aalzahrani@kfshrc.edu.sa (A.A.); alialqahtani@kfshrc.edu.sa (A.A.);; 2 Department of Pathology, King Faisal Specialist Hospital & Research Centre, Riyadh 11211, Saudi Arabia; 3 Innovation and Research, King Faisal Specialist Hospital & Research Centre, Riyadh 11211, Saudi Arabia; 4 College of Medicine, Al-Faisal University, Riyadh 11533, Saudi Arabia

**Keywords:** chemoradiotherapy, rectal cancer, gemcitabine, immune

## Abstract

**Background**: Standard neoadjuvant chemoradiotherapy for locally advanced rectal cancer typically employs capecitabine or 5-fluorouracil. Gemcitabine, an alternative radiosensitizer, has a well-established immunomodulatory effect. The role of preoperative concurrent gemcitabine with radiotherapy on rectal cancer microenvironment and long-term survival has not been fully elucidated. **Methods**: In this phase II clinical trial update and secondary analysis, 40 adult patients with stage T3/T4 or node-positive, non-metastatic rectal cancer received neoadjuvant chemoradiotherapy consisting of external beam radiation (45–54 Gy) with weekly 24-hour infusional gemcitabine (100 mg/m^2^, later 75 mg/m^2^ for toxicity) followed by surgery and adjuvant capecitabine. The protocol was amended to analyse immune cell infiltration pre- and post-treatment using immunohistochemistry. The primary endpoint was pathological complete response (pCR); secondary endpoints included R0 resection rate, toxicity, immune infiltration, disease-free survival (PFS), and overall survival (OS). Results were compared to historical controls treated with capecitabine-based chemoradiation. **Results**: Of the 40 enrolled patients (83% high-risk features), 32 underwent surgery, and 31 were resected. The updated median PFS was 70 months (median follow-up: 87.4 months); median OS was not reached. The estimated 5-year PFS and OS were 54.4% and 67.5%, respectively. Infusional gemcitabine induced significantly higher total immune cell infiltration in resected tumors compared to controls (*p* = 0.026). CD8+ T cell density increased markedly in surgical specimens (*p* = 0.001), and PD-L1+ immune cells rose significantly post-therapy (*p* = 0.032). There was a trend toward increased CD56+ NK cell infiltration. Toxicities and pCR rates aligned with established regimens. **Conclusions**: Neoadjuvant chemoradiotherapy with infusional gemcitabine yields durable survival and robust immune cell infiltration in locally advanced rectal cancer, comparable to modern standards. The immunomodulatory effects of gemcitabine—particularly the enrichment of CD8+ T cells and PD-L1+ immune cells—support further evaluation of combination strategies incorporating immunotherapy to enhance systemic disease control.

## Simple Summary

We assessed immune cell infiltration following chemoradiotherapy in rectal cancer patients enrolled in a completed trial evaluating the effect of gemcitabine-based therapy, compared with the standard 5-fluorouracil–based chemoradiotherapy. We further evaluated dynamic changes in immune cell infiltration by comparing the original diagnostic pre-treatment biopsies with the corresponding surgically resected rectal tumors after treatment. The results show a significantly higher immune cell density in resected tumors from patients treated with gemcitabine-based chemotherapy compared with archived resected tumors from standard 5-fluorouracil-based chemotherapy-treated patients. Specific subsets of immune cells increased in resected tumors compared with pre-treatment biopsies. Interestingly, expression of a targetable immunosuppressive molecule increased, suggesting the possibility of combination therapy.

## 1. Introduction

The approach to managing locally advanced rectal cancer has undergone significant development. Preoperative chemoradiotherapy has supplanted postoperative adjuvant chemoradiotherapy, primarily due to enhanced local control and reduced toxicity [1]. Evidence indicates that neoadjuvant short-course radiation therapy achieves outcomes comparable to those of long-course concurrent chemoradiotherapy [2,3]. More recently, total neoadjuvant therapy (TNT) has demonstrated improved relapse-free survival when compared with standard long-course chemoradiotherapy utilizing capecitabine [4,5]. The question of whether TNT should supplant standard chemoradiotherapy for all patients with localized rectal cancer or be reserved for those with high-risk features remains unresolved. Despite these advancements in rectal cancer management, local recurrence persists at approximately 7% [4], while distant metastasis occurs in about 23% of cases [6].

Immunotherapy with checkpoint inhibitors has enhanced treatment outcomes in various cancer types. Nevertheless, when microsatellite instability-high (MSI-H) tumors are excluded, responses to these agents have generally been suboptimal [7,8]. In rectal cancer, studies indicate that combining immune checkpoint inhibitors with capecitabine and concurrent radiotherapy does not lead to significant improvements in clinical complete response (cCR), pathological complete response (pCR), or neoadjuvant rectal score [9].

We previously conducted a phase II trial of infusional gemcitabine with long-course radiotherapy in patients with locally advanced rectal cancer [10]. Many patients showed inflammatory reactions on imaging and during surgery, leading us to revise our protocol to assess immune cell infiltration pre- and post-treatment. Here, we report these findings on immune infiltration and updated long-term survival data.

## 2. Methods

### 2.1. Patients

In this one-arm phase II clinical trial, adult patients (aged 18 years or older) with histologically confirmed, locally advanced, non-metastatic rectal cancer were considered eligible for inclusion. Eligibility required a clinical tumor stage of T3 or T4, or any T stage with nodal involvement classified as N1-2. Additional criteria included adequate bone marrow, renal, and hepatic function, an Eastern Cooperative Oncology Group (ECOG) performance status of 0–2. All eligible patients were offered to join with the provision of signing a written informed consent. Details regarding eligibility criteria and treatment protocol have been previously published [10]. In summary, pretreatment assessments included a chemistry profile, a complete blood count, carcinoembryonic antigen (CEA) testing, endoscopic rectal ultrasound, computed tomography (CT) scans of the chest, abdomen, and pelvis, magnetic resonance imaging (MRI) of the pelvis, and positron emission tomography (PET) scans.

Patients received external beam radiotherapy at a dose of 1.8 Gy per fraction, given in 25 fractions for a total dose of 45 Gy. An additional boost dose of 5.4 Gy was provided for individuals with T3 lesions, while a boost of 9 Gy was given to those with T4 lesions. Gemcitabine was administered as a 24-h infusion at a dosage of 100 mg/m^2^ weekly, commencing on day one of radiation therapy and continuing for six weeks. On 8 April 2017, the protocol was modified to reduce the gemcitabine dose to 75 mg/m^2^ based on initial toxicity findings.

All patients were scheduled to undergo surgical removal of the primary tumor with total mesorectal excision 10–12 weeks after completing radiotherapy. Adjuvant chemotherapy with capecitabine was administered at a dose of 1250 mg/m^2^ twice daily for 14 days per cycle, over a total of 6 cycles. Follow-up procedures consisted of history and physical examination, complete blood count, chemistry panel, and CEA testing. Patients had follow-ups every three months during the first year, every four months in the second year, and every six months from the third to the fifth year. Additionally, a CT scan of the chest, abdomen, and pelvis was performed annually for five years [10].

On 21 January 2021, the protocol was revised to incorporate evaluation of inflammatory markers (immune cell infiltration) in pre-treatment biopsies and post-therapy specimens, based on observed surgical and radiological findings of local inflammatory reaction.

We have further compared immune cell infiltration in surgically resected tumors from this current trial (Gemcitabine-treated patients) with tumors from a control cohort that received Capecitabine (standard-of-care-control group). The control samples were from a previously published phase 2 trial that employed pre-operative concurrent capecitabine and radiotherapy in locally advanced rectal cancer [11]. Briefly, patients in the control group received a total radiotherapy dose of 50.4 Gy in 28 single daily fractions over 51/2 weeks (5 days per week). Capecitabine was given orally at a dose of 825 mg/m^2^ twice daily throughout the radiotherapy period, starting on day 1, without weekend breaks. This control group of patients was selected as it closely matches the clinical response outcomes (partial or complete response) to allow distinction between the histological effects of chemotherapy and its influence on immune infiltration (Table S1).

### 2.2. Evaluation of Immune Cell Infiltration

Formalin-fixed paraffin-embedded (FFPE) tissue blocks were obtained from pre-treatment biopsies and excised surgical resections. Hematoxylin and eosin (H&E) sections (4 µm) were available from each tissue block as part of routine hospital care for rectal cancer patients. An independent pathologist, blinded to the treatment outcome, scored and interpreted the histological tumor sections.

### 2.3. Immunohistochemistry

Formalin-fixed paraffin-embedded (FFPE) tissue blocks of biopsy or surgical sections were 4 µm-sectioned, mounted on glass slides and dried in an oven at 60 °C for 1 hour. Immunohistochemistry was performed using a fully automated Ventana Benchmark Ultra (Ventana/Roche) system. The antigen retrieval was performed using the ULTRA CC1 solution (Ventana), and the immunostaining was performed using Ventana’s validated reagents and ready-to-use primary antibodies (Table S2). The diaminobenzidine (DAB) substrate-based Ultraview kit was used for signal visualization.

Among the 31 patients who underwent surgery, archived surgical tissue blocks were available from 25 patients, and biopsy blocks were available for 20 (paired biopsy and surgical tissues were available for only 17 patients). Characteristics of the 31 patients is described in Table S1.

TIL was scored by an anatomical pathologist (HA). H&E sections of tissues from gemcitabine-treated patients were compared to a control group that received capecitabine as neoadjuvant chemotherapy.

As adapted from Salgado et al. [12] and Loi et al. [13], total lymphocyte infiltration was evaluated using a semi-quantitative 4-tier scoring method. The score was based on the percentage of tumor-infiltrating lymphocytes (TILs) within the tumor field, as assessed by morphology. A score of 1 was assigned for absent or rare TILs (<10% of the field), while scores of 2 (mild), 3 (moderate), and 4 (severe) corresponded to 10–30%, 30–50%, and >50% of the field, respectively.

For the subpopulations of immune cells, including CD8+ TILs, CD56+ TILs (NK cells), PD-L1+ TILs, and CD33+ MDSCs, the actual percentage of cells occupying the tumor field was recorded directly, without applying the 4-tier scoring system, due to the small proportion of these subpopulations as compared to the overall TIL.

### 2.4. Endpoints and Statistical Analysis

The primary endpoint for the trial was pCR with the intention to treat. Secondary endpoints included R0 resection rate, toxicity evaluation and immune cell infiltration before and after chemoradiotherapy. Progression-free and overall survival were exploratory endpoints. Patients who die, progress before or develop severe toxicity prohibiting surgery were considered non-pCR. The sample size was calculated using the optimal 2-stage design. This call was for evaluating the first 15 recruited patients. If two or less achieved pCR, then the trial would be terminated. In the second stage, with a total of 35 patients to be recruited, the trial would be considered negative if ≤6 patients achieved pCR. Additionally, the protocol called for termination if 30% or more patients developed grade 4 or 5 toxicity.

Toxicity was scored according to the National Cancer Institute’s Common Toxicity Criteria for Adverse Events, version 4. Progression-free survival was calculated from the date of starting chemoradiotherapy until the date of progression, recurrence, or death. Overall survival was calculated from the date of starting chemoradiotherapy until death or the last follow-up. The Kaplan–Meier method was used to calculate survival time.

The protocol was conducted in accordance with the principles of the Declaration of Helsinki and the Good Clinical Practice guidelines. The study was approved by the hospital’s research ethics committee and registered at clinicaltrials.gov under the number NCT02919878. All patients signed written informed consent. Paper-based data on patients were protected in a locked cabinet. Electronic data were saved in password-protected files. Only the principal investigator and clinical staff had access to patient confidential information.

### 2.5. Statistical Analysis

Survival curves were summarized using a Kaplan–Meier estimator (SPSS, version 24). Curves were generated using the IBM SPSS, version 24software (Armonk, NY, USA). OS was defined as the time to death from any cause. Numerical variables between two independent groups were compared using a two-tailed unpaired Student’s t-test: gemcitabine-treated patients (*n* = 31), with pre-treatment available biopsy samples (*n* = 20) versus surgical resection samples (*n* = 25). For patients with paired biopsy and surgical resection samples (*n* = 17), versus the control group treated with capecitabine (*n* = 30), comparisons were made using a two-tailed paired Student’s t-test. Statistical analysis was performed using GraphPad Prism 5.0 software (La Jolla, CA, USA).

## 3. Results

### 3.1. Treatment Outcome

Forty patients were recruited between 9 March 2015 and 9 October 2018, including 33 with one or more high-risk features (T4, N2, threatened margin, or extramural venous invasion). Table 1 illustrates the patients’ characteristics. One patient died before completing preoperative chemoradiotherapy; all others completed the planned treatment. The median radiation dose was 5040 cGy (range, 4500–5500).

Three patients did not undergo pre-surgical evaluation, including one who died (listed above), and 2 withdrew consent. Among the remaining 37 patients, radiological assessment for downstaging revealed the following: T3 in 11, T2 in 5, and T4 in 6 patients. Additionally, 13 and 5 patients had N1 and N2, respectively. Unfortunately, five patients progressed with distant metastasis while on chemoradiotherapy as follows: one in the retroperitoneal node, one with multiple unresectable liver metastases, and 3 with resectable liver metastases.

Altogether, eight patients did not undergo surgery, including 5 who withdrew consent, 2 who progressed to unresectable disease, and 1 who died during chemoradiotherapy. On the other hand, surgery was performed in 32 patients: 11 abdominoperineal resections, 16 anterior resections, 2 anterior resections with liver metastasectomy, 1 abdominoperineal resection with liver metastasectomy, and 1 proctocolectomy with ileoanal anastomosis. One patient was deemed unresectable at laparotomy due to peritoneal carcinomatosis. Figure S1 represents the flow diagram of the above.

**Figure 1:**
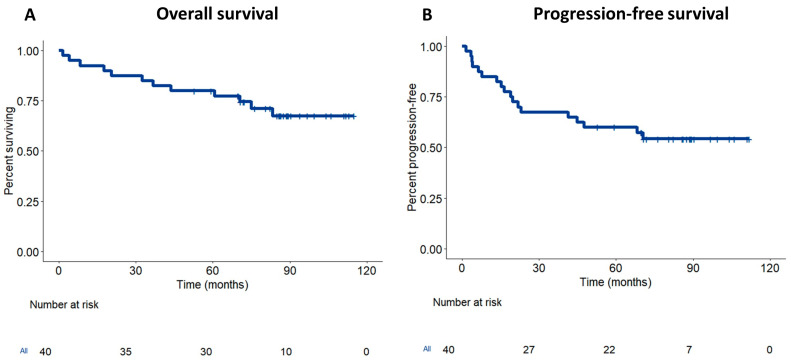
Survival of locally advanced rectal cancer treated with neoadjuvant gemcitabine. Kaplan–Meier survival curves showing overall survival (OS) (**A**) and disease-free survival (DFS) (**B**) of rectal cancer treated with neoadjuvant gemcitabine and radiotherapy.

The data cutoff for the updated analysis was 30 October 2024. At the time of data lock, thirteen patients had progressed or developed recurrence, and eleven died. Notably, 2 of the low-risk group (29%) and 11 of the high-risk group (33%) developed recurrence. The median follow-up for this analysis was 87.4 months (95% CI: 83.5–91.4). The updated median PFS was 70 months, and the overall survival rate was not reached. The estimated 5-year PFS was 54.4% (95% CI: 37.4–68.3%) and OS was 67.5% (95% CI: 49.3–80.3%) (Figure 1).

### 3.2. Immune Cell Infiltration

**Figure 2:**
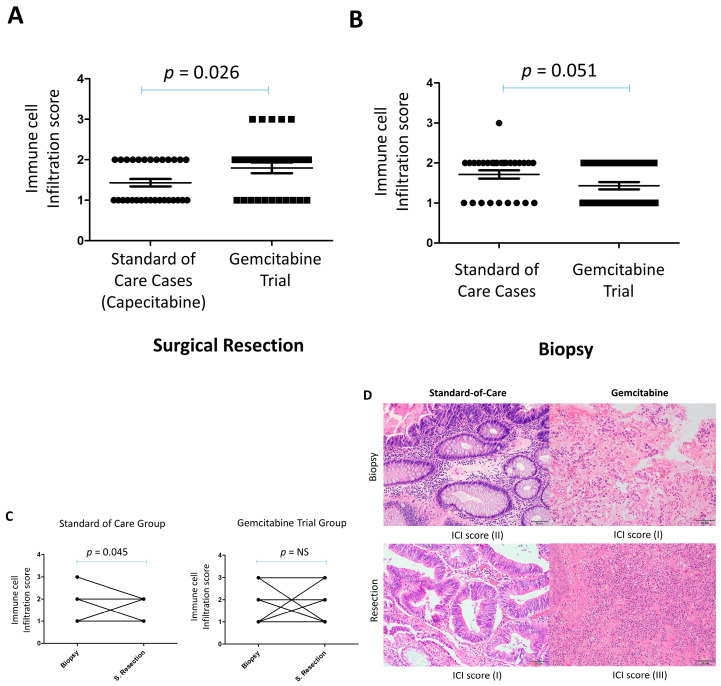
Increased immune cell infiltration in surgical resections of locally advanced rectal cancer following neoadjuvant gemcitabine compared with standard-of-care capecitabine. (**A**) Immune cell infiltration scores in surgical resection specimens. (**B**) Immune cell infiltration scores in diagnostic pretreatment biopsies. (**C**) Paired comparisons of immune cell infiltration scores between biopsies and surgical resections for each patient treated with either gemcitabine (GEM) or capecitabine. (**D**) Representative H&E-stained sections illustrating immune infiltration in paired biopsy and surgical resection samples. NS = Not significant. Scale bar = 50 µm).

The immune cell infiltration in the surgically resected tumors of gemcitabine-treated patients was significantly higher (*p* = 0.026) than in the control group (Figure 2A). This higher immune infiltration in the surgically resected tumors was evident, despite the trend of lower immune cell infiltration in the original tumor biopsy of gemcitabine-treated patients compared to the control group (*p* = 0.051) (Figure 2B). The immune cell infiltrate in the gemcitabine-treated patients consisted of a mixture of plasma cells, lymphocytes, eosinophils, and neutrophils. Interestingly, plasma cells were most notable in cases with high immune cell infiltration (score 3), which was observed in gemcitabine-treated patients but not in the control group.

We then compared the immune infiltration between the paired surgically resected tumors and the original pre-treatment biopsy at diagnosis. While there was no significant change in immune cell infiltration between the resected tumors and pre-treatment tissue biopsies in this trial, there was a decrease in immune cell infiltration in the resected tissues compared to pre-treatment biopsies in the control group (*p* = 0.045) (Figure 2C). Interestingly, a change was observed in the proportion of certain types of immune cells between resected tumors and the pre-treatment biopsy. Thus, while lymphocytes and plasma cells tended to increase in resected tumors, neutrophils and eosinophils were present with lymphocytes in the pre-treatment biopsies (Figure 2D).

### 3.3. Gemcitabine Significantly Increased CD8+ and PD-L1-Positive Infiltrating Immune Cells

**Figure 3:**
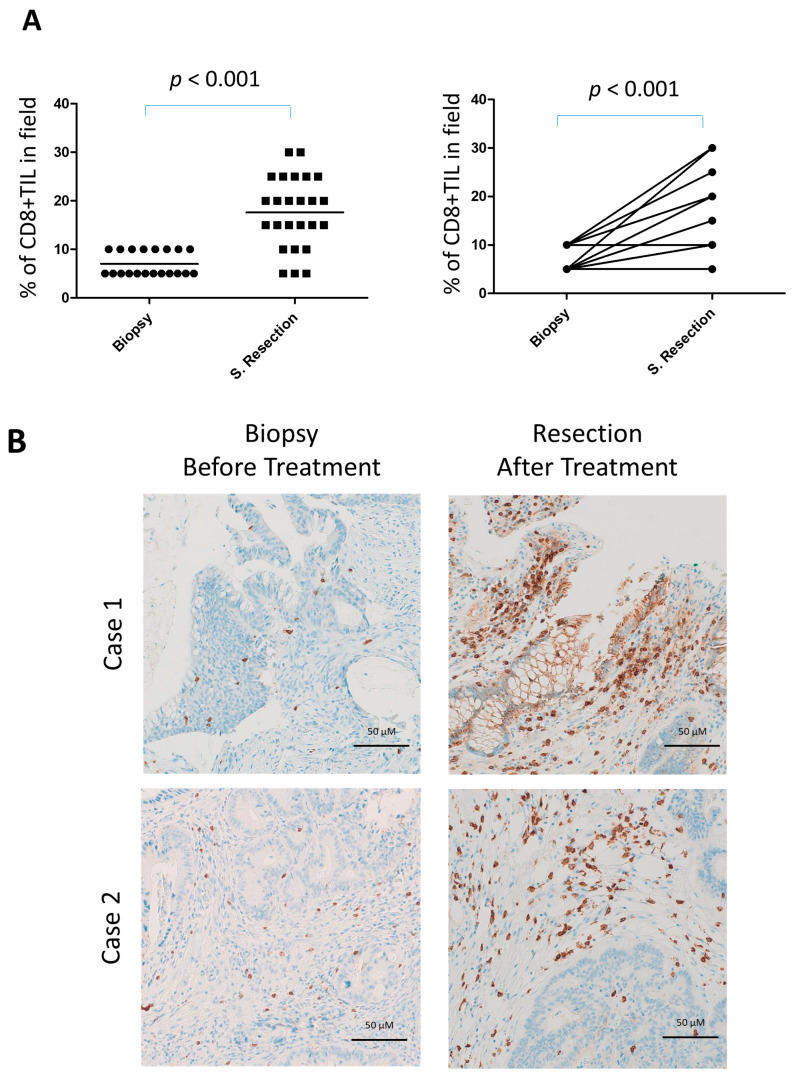
Increased CD8+ lymphocyte infiltration in surgical resections of locally advanced rectal cancer following neoadjuvant gemcitabine. (**A**) Quantification of CD8+ tumor-infiltrating lymphocytes (TILs) in surgical resections (*n* = 25) compared with diagnostic pretreatment biopsies (*n* = 20) (left) and in the subset of cases with available paired biopsy and surgical resection samples (*n* = 17) (right). (**B**) Representative immunohistochemical staining for CD8 in paired biopsy and surgical resection samples.

Using immunohistochemistry, we examined specific subpopulations of lymphocytes in the tumor tissues of this trial. There was a significant increase in CD8^+^ T-cell infiltration in surgically resected tumors compared with pretreatment biopsies (*p* < 0.001; Figure 3A,B). Analysis of paired tissue samples further confirmed this finding, showing a highly significant elevation in CD8^+^ T-cell density in post-surgical specimens relative to their matched pretreatment biopsies (Figure 3A, right).

There was a trend of an increase in CD56+ Lymphocytes (NK cells) in the resected tumor compared to the pre-treatment biopsy, although it did not reach significance (Figure S2).

**Figure 4:**
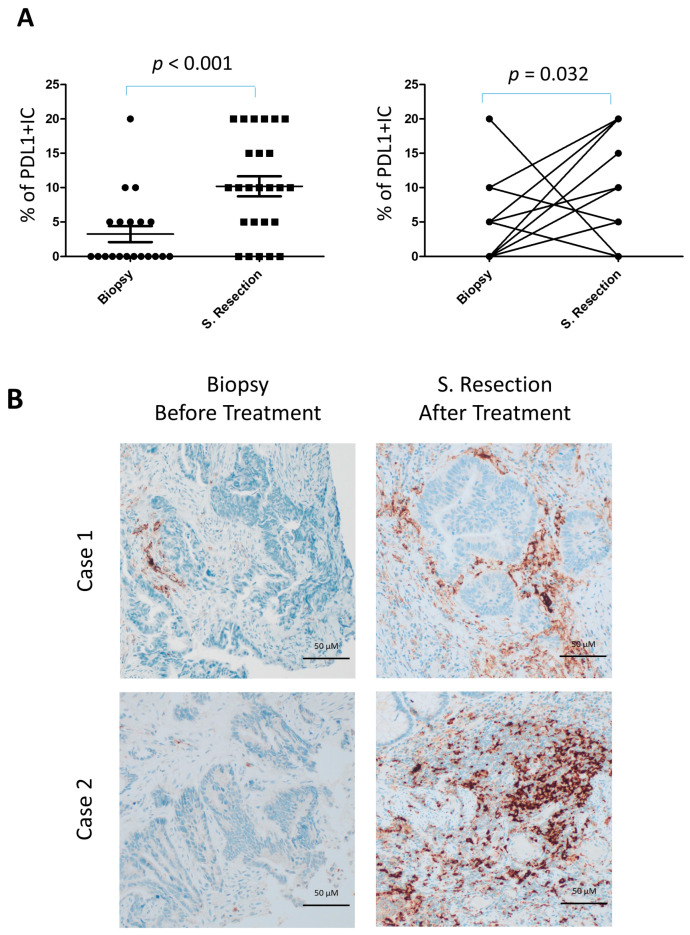
Increased PD-L1+ lymphocyte infiltration in locally advanced rectal cancer following neoadjuvant gemcitabine. (**A**) Quantification of PD-L1+ tumor-infiltrating lymphocytes (TILs) in resection specimens (*n* = 25) versus diagnostic pretreatment biopsies (*n* = 20) (left), and in matched biopsy–resection pairs (*n* = 17) (right). (**B**) Representative immunohistochemical staining of PD-L1 in paired biopsy and resection samples.

PD-L1 expression in immune cells was detected in 19 of 25 surgical resections (76%) compared to 7 of 20 biopsies (35%). The frequency of PD-L1 positivity was significantly higher in surgical specimens (*p* < 0.001), even when the actual percentage scores were analyzed (Figure 4A,B). In paired comparisons between biopsies and surgical resections from the same patients, PD-L1 expression in immune cells was also significantly higher in resections (*p* = 0.019), both when the absolute number of infiltrating cells was considered and when assessed by scoring (*p* = 0.012) (Figure 4A, right). In contrast, PD-L1 expression in tumor cells was rare, being observed in only one case of surgical resection and one biopsy.

We also looked for CD33**^hi^** as a marker of myeloid-derived suppressor cells (MDSC). There were no significant differences between biopsies and surgical resections, even when the paired biopsy/surgical tissues from the same patients were compared (Figure S3).

Altogether, gemcitabine increased total immune infiltration as compared to capecitabine. Specifically, there was an increase in CD8+ T-cells and CD56+ NK cells. Moreover, there was an increase in PD-L1+IC infiltration after gemcitabine-based chemoradiotherapy.

## 4. Discussion

Our updated median PFS of 70 months, together with the fact that median OS has not yet been reached, underscores the durability of control in a proportion of cases, despite the predominance of high-risk features among our participants.

These survival and recurrence figures fall within the range recently reported for modern neoadjuvant approaches. For example, 5-year OS and PFS rates from the RAPIDO trial (standard long-course chemoradiotherapy arm) and the UNICANCER-PRODIGE 23 trial (total neoadjuvant FOLFIRINOX + chemoradiotherapy) are in the 65–75% range for high-risk cohorts, with local recurrence now consistently under 10%, but distant metastasis rates persistently above 20% [14,15]. Our recurrence profile—a higher proportion of distant versus local recurrence—mirrors these recent studies, confirming the ongoing challenge of systemic disease despite excellent primary tumor control.

Importantly, our updated cohort also demonstrates clinical outcomes comparable to recent real-world and trial data for both standard capecitabine-based chemoradiation and TNT regimens, despite constituting a predominantly high-risk Saudi cohort [16]. Our pathological complete response (pCR) rates and toxicity profile remain consistent with prior reports, and the durability of long-term disease control supports the feasibility of the gemcitabine-based protocol in select patient populations. Nonetheless, as with other contemporary series, the persistence of distant relapse highlights an area of unmet need, underscoring arguments for intensified or targeted systemic therapy in future strategies. Apart from 5 patients withdrawing consent, only 4 patients did not go for curative resection, constituting around 11% of enrolled patients, which is in line with advanced rectal cancer in general.

On the other hand, immunotherapy with immune checkpoint inhibitors (ICIs) is becoming the backbone of management for several types of cancer. Unfortunately, the efficacy of ICIs in microsatellite-stable (MSS) colorectal cancer patients, which constitute up to 80–85% of colorectal cancer, is still poor. Moreover, the majority of data is related to colon cancer, while data on rectal cancer is more limited. In this manuscript, we report, for the first time, on immune infiltration in rectal cancer patients before and after gemcitabine-based chemoradiotherapy, although we cannot confirm that all are MSS as MSI/MMR testing was not standard practice at that time. We have demonstrated that gemcitabine resulted in higher immune infiltration than standard-of-care therapy. Importantly, gemcitabine modulated the type of immune cell infiltration, promoting CD8+ T cells and NK cells infiltration. Notably, there was an increase in PD-L1-positive immune cells following gemcitabine-based chemoradiotherapy.

Gemcitabine, a pyrimidine antimetabolite, kills cancer cells by apoptosis that is induced by early termination of DNA synthesis. Importantly, there is mounting evidence that gemcitabine has immunomodulatory properties beyond its cytotoxic effect on cancer cells [17]. Gemcitabine alters the composition of the tumor secretome by increasing the expression of a CCL/CXCL family cytokine [18], leading to a change in the immune cell composition of tumor tissue. Several studies have observed a decrease in the percentage of T-reg cells in patients’ peripheral blood after treatment with gemcitabine [19,20]. T-reg cells inhibit the function of effector cytotoxic lymphocytes, and their decrease after gemcitabine treatment is expected to boost the anti-tumor immune response [21]. Moreover, gemcitabine inhibits myeloid-derived suppressor cells (MDSCs) in mouse models of lung and breast cancers [22,23]. In this trial, there was a low level of MDSCs in tumor biopsies before treatment, and no significant changes were observed after GEM therapy.

In the tumor microenvironment of a mouse model of pancreatic cancer, gemcitabine increased the number and cytotoxicity of natural killer (NK) cells [24]. Moreover, treatment with low doses of gemcitabine has been shown to enhance the activity of natural killer cells in lung cancer [25]. In the current trial, which also used a low dose of gemcitabine, we observed a significant increase in NK cells in surgical resection specimens compared to the original tumor biopsy, although the data for paired biopsy/surgical tissues were not statistically significant. In another study using a mouse model of breast cancer, gemcitabine and Cyclophosphamide increased both cytotoxic CD8+ T cells and NKT cells in the tumor [26]. No such data were demonstrated in rectal cancer. We have shown in this trial that gemcitabine, in combination with radiotherapy, significantly increases tumor infiltration by CD8+ T cells.

PD-L1 expression in tumor tissues was uncommon (4–6%) in both the original biopsy and surgical resection. This is consistent with previous reports. Jomrich et al. reported a lack of PD-L1 expression in the rectal cancer studied [27] while Coussement et al. reported a 4% and 15% expression in surgical and biopsy rectal tissues, respectively [28].

The expression of PD-L1 on TIL can lead to their suppression by binding to CD80 (B7-1) on other cells, like tumor cells or other infiltrating immune cells (in trans) [29]. Alternatively, PD-L1 on TIL can bind to CD80 on the same cell (in cis), making PD-L1 inaccessible to PD-1 binding, leading to T-cell activation [30]. While the outcome of these interactions is complex and affects T cells differently depending on the context, there is evidence that the “in trans” effect of PD-L1 on TIL is more dominant than the “in cis” effect [29].

It is believed that combining immune checkpoint inhibitors (ICPs) with chemotherapy or other treatment modalities could improve the response of colorectal cancer to ICPIs. However, the appropriate chemotherapeutic agent to combine with ICPs is not well defined. In this study, we have shown that gemcitabine can immunomodulate the immune cell infiltration in rectal cancer. This response would likely be improved if treatment is combined with immunotherapy. In fact, we have demonstrated a significant increase in PD-L1-positive immune cells in surgical resections after gemcitabine therapy compared to the original tumor biopsy, although we cannot confirm that this specific to gemcitabine as the historical standard-of-care patients control group were not tested for PD-L1 expression. The increased PD-L1 expression after CRT may be secondary to factors such as treatment-induced tissue damage, cytokine release, and immune activation [31]. Nonetheless, PD-L1 upregulation post therapy makes it possible to test a combination with anti-PD-L1 therapy to boost the gemcitabine immunomodulatory effect and enhanced systemic disease control. In fact, in a model of nasopharyngeal carcinoma, gemcitabine has been shown to adversely promote the expression of PD-1 in NK cells and its ligand (PD-L1) in cancer cells through the NF-κB pathway [32]. Conversely, PD-L1/PD-1 checkpoint blockade has been shown to enhance the cytotoxicity of NK cells. Whether this will be the case in rectal cancer needs further investigation.

While we report improved survival outcomes and evidence of an immunomodulatory role of gemcitabine in rectal cancer, this study has limitations, including the small sample size and the lack of information on MSI/MMR status, as well as the lack of comparison with CD8+ and PD-L1+ TIL in the historical standard-of-care control group.

## 5. Conclusions

In summary, neoadjuvant chemoradiotherapy with infusional gemcitabine demonstrates durable survival outcomes and significant enhancement of immune cell infiltration, particularly CD8+ T cells and PD-L1+ lymphocytes, in locally advanced rectal cancer. These findings suggest that gemcitabine not only supports effective tumor control but also modulates the tumor microenvironment in ways potentially synergistic with immunotherapy. Despite a high proportion of high-risk cases in our cohort, disease control and survival rates were favorable, comparable to contemporary protocols. The persistent challenge of distant relapse underscores the need for advancing systemic management strategies. Our data support further exploration of gemcitabine-based chemoradiotherapy in combination with immune checkpoint inhibitors to potentially amplify antitumor immunity and improve long-term clinical outcomes.

## Figures and Tables

**Table 1:** Characteristics of 40 patients treated with preoperative concurrent radiation and infusional gemcitabine.

Characteristic	Number = 40 (%)
Age, Median (range)	60.5 (38–83) years
Male sex	26 (65)
Performance status	
0	11 (27)
1	25 (63)
2	4 (10)
Histological grade	
Well Differentiated	5 (13)
Moderately Differentiated	30 (75)
Poorly Differentiated	3 (7)
Not determined	2 (5)
RAS status	
KRAS/NRAS mutant	16 (40)
Not amplified	12 (30)
Low hemoglobin	17 (43)
Pre-treatment colostomy	11 (28)
Clinical T stage	
T2	2 (5)
T3	24 (60)
T4	14 (35)
Clinical *N* stage	
N0	9 (23)
N1	18 (45)
N2	13 (32)
Clinical stage grouping	
IIA	3 (8)
IIIB	27 (67)
IIIC	10 (25)
* EMVI	27 (67)
Threatened Margin	23 (57)
Risk class	
Low risk	7 (18)
High risk	33 (83)
Distance from anal verge	
<5 cm	16 (40)
5–10 cm	21 (53)
>10 cm	3 (7)
Tumor arising below the Levator Ani	11 (28)

* EMVI: Extra-mural vein invasion.

## Data Availability

The analyzed datasets in this study are included in this published article (or Supplementary Files) and available from the corresponding author upon reasonable request.

## Supplementary Materials

The following supporting information can be downloaded at: https://www.mdpi.com/article/10.3390/cancers17243963/s1. Figure S1: Trial recruitment diagram; Figure S2: Neoadjuvant gemcitabine enhances NK cell infiltration; Figure S3: The effect of neoadjuvant gemcitabine on MDSC infiltration; Table S1: The Characteristics of patients analyzed by H&E and immunohistochemistry; Table S2: List of Antibodies used.
