# Supplementary material for: Neoadjuvant Chemoradiotherapy for Locally Advanced Rectal Cancer Using Infusional Gemcitabine: Immune Cell Infiltration Analysis and Updated Survival
Source: Cancers (Basel). 2025 Dec 12;17(24):3963. doi: 10.3390/cancers17243963 (PMC12730363; doi:10.3390/cancers17243963)
Supplement: Supplementary file 1 [file cancers-17-03963-s001.zip › cancers-3976266-supplementary.pdf]

Supplementary Materials: Neoadjuvant Chemoradiotherapy for Locally Advanced Rectal Cancer Using Infusional Gemcitabine: Immune Cell Infiltration Analysis and Updated Survival

Shouki Bazarbashi, Hadeel AlManea, Ali Aljubran, Ahmed Alzahrani, Ali Alqahtani, Fahad Almugbel, Muhammad Shazad Rauf and Hazem Ghebeh

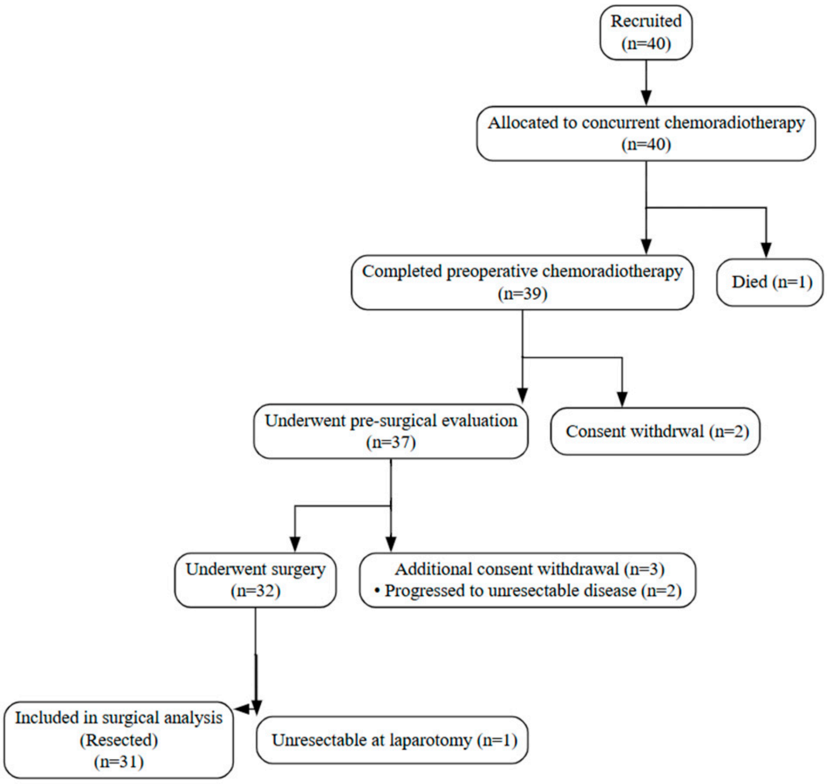

Figure S1. Trial recruitment diagram. Schematic diagram showing the recruitment status.

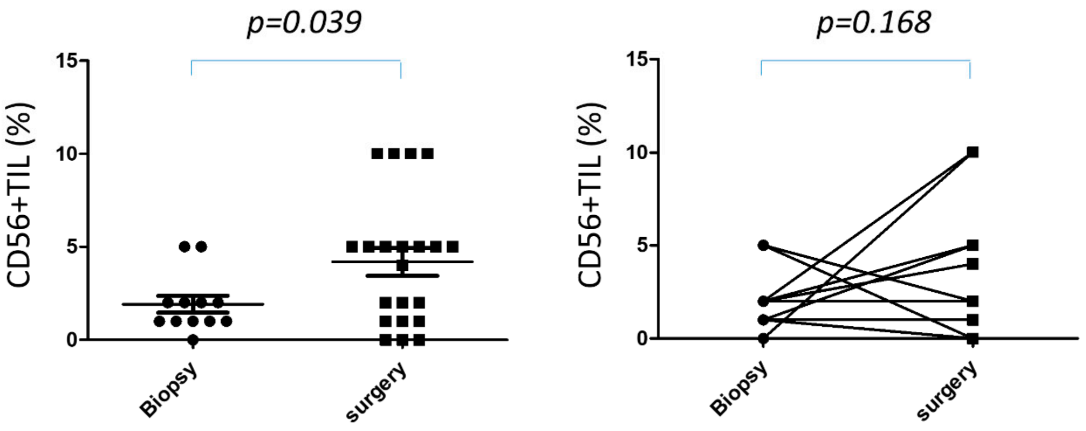

Figure S2. Neoadjuvant gemcitabine enhances CD56^{+} lymphocyte (NK cell) infiltration in locally advanced rectal cancer. (A) CD56^{+} NK cell counts in resection specimens (n= 25) compared with diagnostic biopsies (n = 20) (left), and in paired biopsy-resection samples from the same patients (n = 17) (right).

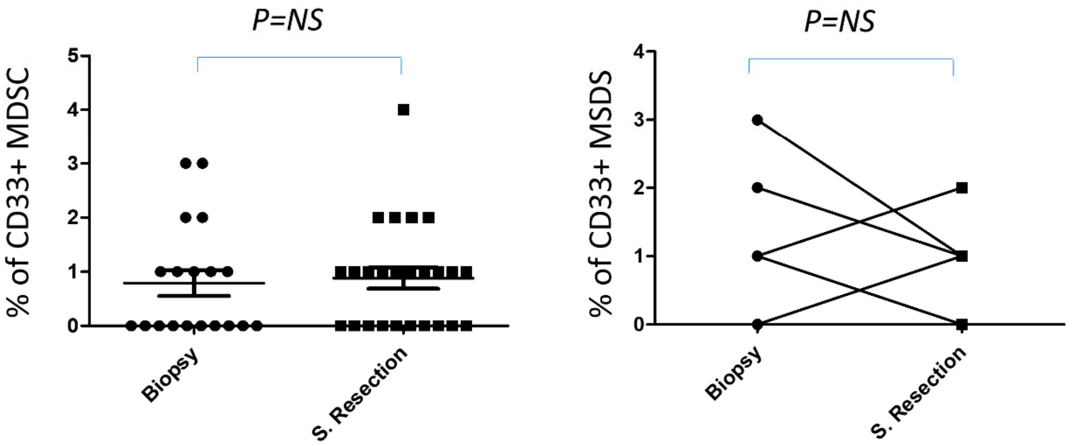

Figure S3. The effect of neoadjuvant gemcitabine on CD33+ immune cells (MDSC) infiltration in locally advanced rectal cancer. (A) CD33+ MDSC counts in resection specimens (n = 25) compared with diagnostic biopsies (n = 20) (left), and in paired biopsy-resection samples from the same patients (n = 17) (right).

Table S1. Characteristics of patients analyzed by H&E and immunohistochemistry in both the Gemcitabine trial group and the standard of care control group.

Characteristic	Gemcitabine Group n=31 (%)	Control Group n=30
Age, Median (range)	57 (38–74) years	58 (36–79)
Male sex	19 (61)	20(67)
Performance status		
0	8 (26)	11 (41)
1	22 (71)	15 (55)
2	1 (3)	1 (4)
Missing	0	3 (10)
Histological grade		
Well Differentiated	5 (16)	1 (3)
Moderately Differentiated	23 (74)	27 (90)
Poorly Differentiated	1 (3)	0
Not determined	2 (7)	2 (7)
RAS status		
KRAS/NRAS mutant	13 (42)	15 (50)
Not amplified	8 (26)	10 (33)
Missing	0	5 (17)
Low hemoglobin	11 (36)	12 (40)
Pre-treatment colostomy	11 (36)	5 (17)
Clinical T stage		
T2	0	6 (20)
T3	20 (65)	21 (70)
T4	11 (36)	3 (10)
Clinical N stage		
N0	3 (10)	3 (10)
N1	17 (55)	14 (47)
N2	11 (36)	13 (43)
Clinical stage grouping		
II	3 (10)	3 (10)
III	28 (90)	27 (90)
*EMVI	20 (65)	6 (20)
Threatened Margin	17 (55)	12 (40)
Risk class		
Low risk	19 (61)	16(53)
High risk	12 (39)	14(47)
Distance from anal verge		
< 5 cm	11 (36)	5 (17)
5–10 cm	17 (55)	17 (56)
> 10 cm	3 (10)	6 (20)
Missing	0	2 (7)

*EMVI: Extra-mural vein invasion.

Table S2. List of Antibodies used

Target	Clone	Source	Cat#
CD33	PWS44	Cell Marque Antibodies	133M-18
PD-L1	SP263	Ventana	07208162001
CD8	SP57	Ventana	05937248001
CD56	MRQ-42	Ventana	06433359001
